# The Slow Dynamics of Intracellular Sodium Concentration Increase the Time Window of Neuronal Integration: A Simulation Study

**DOI:** 10.3389/fncom.2017.00085

**Published:** 2017-09-20

**Authors:** Asaph Zylbertal, Yosef Yarom, Shlomo Wagner

**Affiliations:** ^1^Department of Neurobiology, Institute of Life Sciences, The Hebrew University and the Edmond and Lily Safra Center for Brain Sciences Jerusalem, Israel; ^2^Sagol Department of Neurobiology, University of Haifa Haifa, Israel

**Keywords:** sodium dynamics, pyramidal cells, purkinje cells, mitral cells, neuronal modeling, sodium-potassium-exchanging ATPase, sodium-calcium exchanger

## Abstract

Changes in intracellular Na^+^ concentration ([Na^+^]_i_) are rarely taken into account when neuronal activity is examined. As opposed to Ca^2+^, [Na^+^]_i_ dynamics are strongly affected by longitudinal diffusion, and therefore they are governed by the morphological structure of the neurons, in addition to the localization of influx and efflux mechanisms. Here, we examined [Na^+^]_i_ dynamics and their effects on neuronal computation in three multi-compartmental neuronal models, representing three distinct cell types: accessory olfactory bulb (AOB) mitral cells, cortical layer V pyramidal cells, and cerebellar Purkinje cells. We added [Na^+^]_i_ as a state variable to these models, and allowed it to modulate the Na^+^ Nernst potential, the Na^+^-K^+^ pump current, and the Na^+^-Ca^2+^ exchanger rate. Our results indicate that in most cases [Na^+^]_i_ dynamics are significantly slower than [Ca^2+^]_i_ dynamics, and thus may exert a prolonged influence on neuronal computation in a neuronal type specific manner. We show that [Na^+^]_i_ dynamics affect neuronal activity via three main processes: reduction of EPSP amplitude in repeatedly active synapses due to reduction of the Na^+^ Nernst potential; activity-dependent hyperpolarization due to increased activity of the Na^+^-K^+^ pump; specific tagging of active synapses by extended Ca^2+^ elevation, intensified by concurrent back-propagating action potentials or complex spikes. Thus, we conclude that [Na^+^]_i_ dynamics should be considered whenever synaptic plasticity, extensive synaptic input, or bursting activity are examined.

## Introduction

In modeling the electrical behavior of neurons, the intracellular and extracellular concentrations of the major ions (except Ca^2+^) are typically taken as constant, due to two assumptions: (a) Existence of effective regulating mechanisms that maintain homeostatic conditions; (b) The total number of ions that flow across the membrane during electrical activity is rather small, either because the current is weak or lasts for a short time. Ionic concentration changes have been taken into account in sporadic cases such as [Cl^−^]_i_ fluctuations, which may result in the reversal of GABA_A_ receptor-mediated currents (Wagner et al., 1997; Ben-Ari, 2002), or [K^+^]_o_, which can increase as a result of intense population activity, leading to initiation of seizures (Moody et al., 1974; Yarom and Spira, 1982; Cressman et al., 2009).

In the few studies where changes in neuronal [Na^+^]_i_ were examined, it was used to detect and analyze Na^+^ conductance hot-spots (Fleidervish et al., 2010) or as part of point neuron modeling in the context of seizures or bursting (Cressman et al., 2009; Barreto and Cressman, 2011). Forrest et al. (2012) provided a simplified model of changes in [Na^+^]_i_ in a cerebellar Purkinje cell in order to examine the effect of Na^+^-K^+^ pump current. The study of [Na^+^]_i_ changes and their consequences is significantly more common in the context of cardiac myocyte modeling, where Na^+^ is treated as an intracellular messenger (Swietach et al., 2015).

[Na^+^]_i_ dynamics are governed by Na^+^ influx, pumping, and longitudinal diffusion. In the present study, the primary sources of Na^+^ influx are voltage-dependent Na^+^ conductance (either transient or persistent) and glutamatergic synaptic conductance. Contrary to Ca^2+^, longitudinal diffusion is an important factor in [Na^+^]_i_ dynamics, since Na^+^ is free to diffuse in the cell without buffering. Moreover, at short time scales, Na^+^ diffusion seems to be the major contributing factor to changes in [Na^+^]_i_ (Fleidervish et al., 2010). Due to the prominent role of longitudinal diffusion, [Na^+^]_i_ dynamics are highly dependent on the geometry of the compartment in question and the availability of diffusion sinks in the adjacent compartments. In some cases (e.g., thin processes and/or lack of diffusion sinks), [Na^+^]_i_ dynamics may be several orders of magnitude slower than the dynamics of membrane potential or [Ca^2+^]_i_, thus allowing prolonged temporal integration, or “memory,” of past inputs or activity (Forrest et al., 2012; Zylbertal et al., 2015). Conversely, the relative rapid diffusion of Na^+^ entails that its concentrations in the sub-membrane area and the core of the compartment are in equilibrium.

The main Na^+^ extrusion mechanism is the electrogenic Na^+^-K^+^ pump, which transports two K^+^ ions into the cell for every three Na^+^ ions it transports out of the cell, thus producing net outward current using energy from ATP hydrolysis (Carafoli, 1991). For every unit of charge introduced into the cell as Na^+^ inward current, a net 1/3 unit of charge will contribute to an outward current carried by the Na^+^-K^+^ pump. This outward current exerts a variable effect on the membrane potential and thus on neuronal information processing (Forrest, 2014). Another source for Na^+^-dependent outward current may be Slick and Slack K^+^ channels (Kaczmarek, 2013), which will not be discussed here.

Another important consequence of changes in [Na^+^]_i_ are variations in Na^+^ Nernst potential (E_Na_). Similarly to [Na^+^]_i_, this value is usually regarded as constant, although as we demonstrate below, bouts of spiking activity or synaptic inputs are sufficient to shift it enough to cause significant spike amplitude adaptation and apparent synaptic depression.

Lastly, [Na^+^]_i_ affects Ca^2+^ dynamics, since the Na^+^-Ca^2+^ exchanger, an important Ca^2+^ extrusion mechanism, is powered by the trans-membrane Na^+^ concentration gradient (Blaustein and Lederer, 1999). Changes in this gradient reduce or even reverse the activity of Ca^2+^ extrusion through the exchanger (Schäfer, 2001; Scheuss et al., 2006; Zylbertal et al., 2015). Since Ca^2+^ dynamics are generally faster than Na^+^ dynamics, [Na^+^]_i_ dictates the stable-state [Ca^2+^]_i_, or the level to which [Ca^2+^]_i_ decays. As we demonstrate below, this may have surprising consequences, such as tagging of active synapses by prolonged elevation in [Ca^2+^]_i_.

Here, we present a detailed analysis of [Na^+^]_i_ dynamics and their consequences in three detailed conductance-based models representing three neuronal types: an accessory olfactory bulb (AOB) mitral cell, a cortical layer V pyramidal cell, and a cerebellar Purkinje cell. We do not take into account changes in [K^+^]_o_, which are usually attributed to population activity rather than the behavior of a single cell. Such changes may enhance or reduce the effects we describe, depending on the context.

This modeling was inspired by our experimental and computational analysis of persistent activity and infra-slow bursting in AOB mitral cells (Zylbertal et al., 2015), where we showed that [Na^+^]_i_ dynamics have a critical role in generating both phenomena. Here, we further examine the role of [Na^+^]_i_ dynamics in the AOB mitral cell model, and extend it to other neuronal types using a revised version of previously published pyramidal cell and Purkinje cell models (Hay et al., 2011; Masoli et al., 2015).

These models were chosen because they combine detailed morphology and biophysics. Contrary to the AOB mitral cell model, their geometry was not reduced and represents the original reconstruction of cell morphology. In addition to representing two different neuronal types, these models also diverge in several design choices, stemming from the different goals they were meant to fulfill (see section Methods). For example, the Purkinje cell model contains a detailed axon, Na^+^ channel mechanism, and Ca^2+^ dynamics, while the pyramidal cell model has a realistic and detailed distribution of conductances along its dendritic tree. These differences allowed us to explore a broad range of possibilities for [Na^+^]_i_ dynamics and their consequences, without adhering to any specific design. More broadly, it should be noted that our goal is not to provide accurate predictions for the behavior of each neuronal type we examine, but rather to explore the landscape of possibilities arising from modeling [Na^+^]_i_ dynamics in neurons.

Our results demonstrate three primary pathways by which [Na^+^]_i_ dynamics may influence neuronal computation: (1) prolonged modulation of local EPSP amplitude due to reduction of E_Na_; (2) prolonged activity-dependent hyperpolarization due to increased activity of the Na^+^-K^+^ pump; (3) synaptic tagging by prolonged Ca^2+^ elevation following Na^+^ loading through repeated inputs.

## Methods

All models were implemented in the NEURON simulation environment with Python (Hines and Carnevale, 1997; Hines et al., 2009). All current injections and voltage recordings were done at the soma.

### AOB mitral cell model

For a full description of this model, see Zylbertal et al. (2015, Supporting Information). Briefly, the model was based on experimental measurements and morphological reconstruction of an AOB mitral cell (Myatt et al., 2012; Zylbertal et al., 2015), and included influx, diffusion, and extrusion of Na^+^ and Ca^2+^ (the latter was restricted to the dendritic tuft). Sodium concentration change in each compartment by influx and efflux through the membrane is given by:

$$\begin{array}{l} \frac{d{\left[{\text{Na}}^{+}\right]}_{i}}{dt}=\frac{I_{{\text{Na}}^{+}}}{FV} \end{array}$$

Where $I_{\text{N}{\text{a}}^{+}}$ is the net Na^+^ current (the product of the local current density and the membrane area), *F* is Faraday constant, and *V* is the compartment volume (fully accessible to Na^+^). Na^+^ is free to diffuse longitudinally, and is pumped out of the cell by the Na^+^-K^+^ pump, modeled using a simple kinetic scheme (see below). Ca^2+^ accumulation was modeled similarly: the entire volume of each compartment was accessible to Ca^2+^, yet, it was not free to diffuse. Ca^2+^ buffering and pumping was also modeled using simple akinetic schemes, while the Na^+^-Ca^2+^ exchanger current followed:

$$I_{NCX}=I_{NCX(max)}\frac{{\left[{\text{Na}}^{+}\right]}_{i}^{3}{\left[{\text{Ca}}^{2+}\right]}_{o}e^{\frac{\gamma V_{m}F}{RT}}-{\left[{\text{Na}}^{+}\right]}_{o}^{3}{\left[{\text{Ca}}^{2+}\right]}_{i}e^{\frac{(\gamma -1)V_{m}F}{RT}}}{\left(k_{m(\text{Na})}^{3}+{\left[{\text{Na}}^{+}\right]}_{o}^{3}\right)\left(K_{m(\text{Ca})}+{\left[{\text{Ca}}^{2+}\right]}_{o}\right)\left(1+k_{sat}e^{\frac{(\gamma -1)V_{m}F}{RT}}\right)}$$

(Courtemanche et al., 1998)

Where *I*_*NCX*(*max*)_ is the maximal current, γ is the voltage dependence parameter, *k*_*m*(Na)_ and *k*_*m*(Ca)_ are the Na^+^ and Ca^2+^ dissociation constants, *k*_*sat*_ is the saturation factor, and *R* and *F* are the gas constant and Faraday constant, respectively.

The model assumes the presence of active Na^+^ channels in the apical dendrites and tufts (Ma and Lowe, 2004), as well as non-uniform channel properties across different compartments (Colbert and Pan, 2002). Evolutionary multi objective optimization algorithm (EMOO, Deb, 2001; Bahl et al., 2012) was used first to find a simplified (“lumped”) geometry that would reproduce the passive electrical properties of the detailed geometry. A second EMOO step was used to fit the model parameters, based on recorded electrophysiological and imaging data. Some membrane mechanisms were based upon published models hosted by ModelDB (Mainen and Sejnowski, 1996; Courtemanche et al., 1998; Lazarewicz et al., 2002; Hines et al., 2004; Korngreen et al., 2005). The model code is available online at: https://senselab.med.yale.edu/ModelDB/ShowModel.cshtml?model=185332.

### Layer V pyramidal cell model

This is an adaptation of a detailed model developed by Hay et al. (2011). Among the models presented in this paper, we selected the one that included an axon. The original model is based on reconstructed cortical layer V pyramidal cells (Le Bé et al., 2007), and was fitted using the EMOO algorithm based on experimental results derived from step current injection (Le Bé et al., 2007), Ca^2+^ spike statistics (Larkum et al., 1999), and back-propagating action potential properties (Larkum et al., 2001).

We modified the model by first introducing Na^+^ accumulation, diffusion, and a pumping mechanism to all compartments, similarly to the AOB mitral cell model (see above). We used the value of 0.3 μm^2^/ms for the Na^+^ diffusion coefficient, approximately the one measured experimentally in dendrites (Mondragão et al., 2016). In order to account for the apparent effect of dendritic spines, we changed this value to 0.03 μm^2^/ms in some simulations (see Supplementary Information). The Na^+^-K^+^ pump was modeled as in the AOB mitral cell, and was distributed using a similar order of magnitude (in mol/cm^2^: soma - 1·10^−11^; axon - 5·10^−12^; dendrites - 1·10^−15^).

We next replaced the Ca^2+^ dynamics of the original model (simple exponential decay) with buffering, pumping, and Na^+^-Ca^2+^ exchange, as in the AOB mitral cell model. The electrogenic effect of the Na^+^-Ca^2+^ exchanger was removed, since its effect on the membrane potential is already taken into account in the fitting of the original model. We used the Ca^2+^ pump density value from the mitral cell model in the pyramidal cell dendrites, and a density six times higher in its soma and axon. We used the maximal current value of the Na^+^-Ca^2+^ exchanger from the mitral cell model throughout the pyramidal cell, and preserved its parameters. Additionally, we updated the Ca^2+^ channel models, so that the Goldman-Hodgkin-Katz equation, rather than the Nernst equation, is used to infer the Ca^2+^ electromotive force. The spatial resolution of the model was tripled to improve simulation accuracy of diffusional elements. This revised model is available online at: https://senselab.med.yale.edu/ModelDB/showModel.cshtml?model=230326.

### Cerebellar purkinje cell model

This is an adaptation of a detailed model developed by Masoli et al. (2015). The original model is based on a reconstructed dendritic tree and soma of a Guinea pig Purkinje cell (Rapp et al., 1994), to which a detailed description of the axon was later added, including the AIS and three nodes of Ranvier. It incorporates available knowledge on localization and gating of 15 Purkinje cell ionic channels, modeled using either Hodgkin-Huxley or Markovian formalism, as well as detailed Ca^2+^ dynamics with pumping, buffering, and approximation of radial diffusion. Free model parameters (maximal conductance values) were initially set according to estimates taken from the literature (Swensen and Bean, 2003), and later manually fine-tuned to match simulation results with somatically-recorded voltage traces elicited by step-current injections.

Since this model already included detailed Ca^2+^ dynamics, its adaptation was straightforward: We adapted it by introducing Na^+^ accumulation and pumping (as in the pyramidal cell model, using identical pump spatial distribution), and replacing some of the Ca^2+^ pumps with Na^+^-Ca^2+^ exchangers, which were not part of the original model. This was done by setting the maximal Na^+^-Ca^2+^ exchanger current in each compartment according to the compartment pump density (multiplied by 10^8^ mA/mol), and subsequently reducing the pump density by a factor of 10^5^.

### Calculation of Na^+^ entry ratio

We calculated the Na^+^ entry ratio (Carter et al., 2009) in each model by dividing the integral of the global Na^+^ current during an action potential by the minimal charge needed to account for the action potential amplitude (i.e., the product of that amplitude and the membrane capacitance). Effective membrane capacitance was assessed by clamping the model cells soma to two different holding potentials, and measuring the capacitance transient. In the Purkinje cell model, this was done after removing the dendrites, due to the high load they exert on the soma, which results in overestimation of the apparent capacitance.

### Simulation of synaptic currents

Local synaptic conductance was simulated as a sum of onset and offset exponentials, whose time constants represent a compromise between fast AMPA receptor-mediated current kinetics and slow NMDA receptor-mediated current kinetics (Spruston et al., 1995, τ_onset_ = 0.5 ms, τ_offset_ = 10 ms, g_max_ = 5 nS). The Na^+^ and K^+^ components of the conductance were inferred from its reversal potential (E_rev_ = 10 mV) and the resting ionic concentrations. Climbing fiber input was simulated as a concurrent input to all dendritic branches whose diameter is >2.2 μm, with τ_onset_ = 0.5 ms, τ_offset_ = 5 ms, Σg_max_ = 250 nS (Wadiche et al., 2001).

### Realistic distributed input

The pyramidal cell model was used to simulate the effects of stochastic distributed inputs (Hay and Segev, 2015). Thousand pre-synaptic “cortical” spike trains followed Poisson distribution with an average firing rate of 1 Hz. Each of these pre-synaptic sources made five contacts in the pyramidal cell dendrites (for a total of 5,000 synaptic contacts), their location randomly chosen from a uniform distribution. Additional 1,000 pre-synaptic “thalamic” spike trains (having 1 Hz background firing rate and three 2–3 s long episodes of 5 Hz firing rate, representing sensory stimuli) made five contacts at sites chosen from a gamma distribution (*k* = 2.0, θ = 60 μm), resulting in higher density of contacts in proximal regions (Rah et al., 2013). These five contacts were either clustered (all in the same site), or each chosen individually. Excitatory synaptic conductance was modeled as detailed above, with reduced peak conductance (g_max_ = 0.5 nS) and slightly longer decay time (τ_offset_ = 15 ms) to account for NMDA receptor connections.

Since the original Na^+^-K^+^ pump model compensates for Na^+^ leak and background synaptic activity, it had to be adjusted to prevent gradual increase in [Na^+^]_i_. This was done by lowering the [Na^+^]_i_ steady-state value of the pump to a quarter of its original value, and increasing its dendritic density to 50 ·10^−15^ mol/cm^2^.

### Inferring Na^+^-K^+^ pump current as a function of [Na^+^]_i_

The Na^+^-K^+^ pump is simulated according to the following reactions:

$$\begin{array}{l} \left[pump\right]+3[{\text{Na}}^{+}]i\overset{k_{1},\;k_{2}}{\Leftrightarrow }\left[pump\text{Na}\right]\\ \left[pump\text{Na}\right]\overset{k_{3},\;k_{4}}{\Leftrightarrow }\left[pump\right]+3[{\text{Na}}^{+}]o\\ \left[totalpump\right]=\left[pump\right]+[pump\text{Na}] \end{array}$$

Therefore:

$$\begin{array}{l} \;\left(1\right)\;\;I_{pump}=-\frac{F}{3}\cdot \frac{d{\left[{\text{Na}}^{+}\right]}_{i}}{dt}=\frac{F}{3}k_{1}\left[pump\right]{{\left[{\text{Na}}^{+}\right]}_{i}}^{3}\\ \;-k_{2}[pump\text{Na}]\\ \left(2\right)\;\;\frac{d[pump\text{Na}]}{dt}=\left[pump\right]\left(k_{1}{{\left[{\text{Na}}^{+}\right]}_{i}}^{3}+k_{4}{{\left[{\text{Na}}^{+}\right]}_{o}}^{3}\right)\\ \;-\left(\left[totalpump\right]-[pump]\right)\left(k_{2}+k_{3}\right) \end{array}$$

Where *F* is the Faraday constant. Assuming that [*pump*Na] is in equilibrium ($\frac{d\left[pump\text{Na}\right]}{dt}=0$), [*pump*] and [*pump*Na] can be inferred and substituted into (1), yielding:

$$I_{pump}=\frac{F}{3}\cdot [totalpump]\frac{k_{1}k_{3}{{\left[{\text{Na}}^{+}\right]}_{i}}^{3}-k_{2}k_{4}{{\left[{\text{Na}}^{+}\right]}_{o}}^{3}}{k_{1}{{\left[{\text{Na}}^{+}\right]}_{i}}^{3}+k_{4}{{\left[{\text{Na}}^{+}\right]}_{o}}^{3}+k_{2}+k_{3}}$$

### Inferring stable-state [Ca^2+^]_i_ as a function of [Na^+^]_i_

The plasma membrane Ca^2+^ pump is simulated like the Na^+^-K^+^ pump (with different stoichiometry). Summing the rate of the Ca^2+^ pump with the rate of the Na^+^-Ca^2+^ exchanger gives the total rate of Ca^2+^ change:

$$\frac{d{\left[{\text{Ca}}^{2+}\right]}_{i}}{dt}=\frac{I_{NCX(max)}}{F}\cdot \frac{{\left[{\text{Na}}^{+}\right]}_{i}^{3}{\left[{\text{Ca}}^{2+}]\right]}_{o}e^{\frac{\gamma V_{m}F}{RT}}-{\left[{\text{Na}}^{+}\right]}_{o}^{3}{\left[{\text{Ca}}^{2+}\right]}_{i}e^{\frac{(\gamma -1)V_{m}F}{RT}}}{\left(k_{m(\text{Na})}^{3}+{\left[{\text{Na}}^{+}\right]}_{o}^{3}\right)\left(K_{m(\text{Ca})}+{\left[{\text{Ca}}^{2+}\right]}_{o}\right)\left(1+k_{sat}e^{\frac{(\gamma -1)V_{m}F}{RT}}\right)}-[totalpump]\frac{k_{1}\cdot k_{3}{\left[{\text{Ca}}^{2+}\right]}_{i}-k_{2}\cdot k_{4}{\left[{\text{Ca}}^{2+}\right]}_{o}}{k_{1}{\left[{\text{Ca}}^{2+}\right]}_{i}+k_{4}{\left[{\text{Ca}}^{2+}\right]}_{o}+k_{2}+k_{3}}$$

Solving for [Ca^2+^]_i_ when $\frac{d{\left[\text{C}{\text{a}}^{2+}\right]}_{i}}{dt}=0$ (equilibrium) yields a complex expression for stable-state [Ca^2+^]_i_. SymPy Python package (Meurer et al., 2017) was used to generate and evaluate this expression for different values of [Na^+^]_i_.

## Results

### Simulated Na^+^ dynamics in multi-compartmental model cell

To examine the Na^+^ dynamics in the three model cells, we stimulated each of them with a 30 Hz, 2-s long pulse train. The pulse amplitude and duration were adjusted in each cell, so that each pulse would evoke a single action potential, resulting in a 30 Hz firing frequency. In the Purkinje cell model, hyperpolarizing DC injection was used to prevent spontaneous firing.

First, in order to test how realistic the models are in terms of Na^+^ intrusion dynamics, we quantified the sodium entry ratio (SER), i.e., the ratio between overall Na^+^ entry in each spike and the total charge needed to account for the spike amplitude (see Methods, Carter et al., 2009). The cortical pyramidal cells exhibited the most efficient dynamics (SER = 1.76), followed by the AOB mitral cell (SER = 3.6) and the Purkinje cell (SER = 5.6). These values are similar in magnitude and relations to experimentally measured values (Carter et al., 2009), and are slightly higher, due to the underestimation of the Na^+^ current in experiments compared to models.

**Figure 1:**
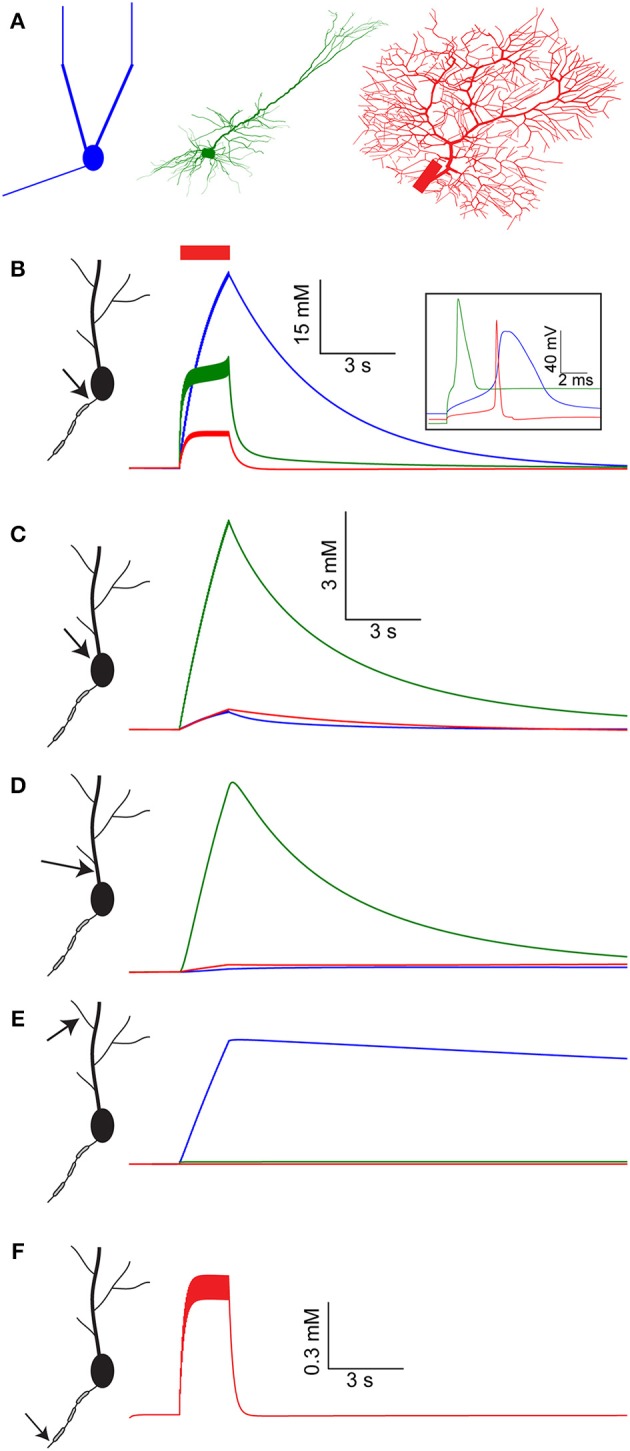
[Na^+^]_i_ dynamics in multiple compartments of the AOB mitral cell, layer V pyramidal cell, and cerebellar Purkinje cell models following a spike train. **(A)** The morphology of the three model cells used: AOB mitral cell with reduced geometry (blue), cortical layer V pyramidal cell (green), and cerebellar Purkinje cell (red). **(B)** [Na^+^]_i_ dynamics in the AIS of the mitral cell model (blue), pyramidal cell model (green), and Purkinje cell model (red) during and after a 30 Hz spike train (red bar). The inset shows the spike shape in the three model cells. **(C–E)** Similar comparison of [Na^+^]_i_ dynamics in the cell somata, proximal dendrite, and distal dendrite. **(F)** [Na^+^]_i_ dynamics in the third node of Ranvier of the Purkinje cell model.

In each of the model cells (Figure 1A), the axon initial segment (AIS) bears (by definition) the highest density of voltage-gated Na^+^ channels, therefore, the largest build-up in [Na^+^]_i_ occurs in this compartment (Figure 1B). The extent and duration of this build-up is determined primarily by the magnitude of Na^+^ influx in each spike and the efficiency of Na^+^ diffusion out of the AIS. Compared to these processes, Na^+^ active extrusion (pumping) is too slow to have a significant effect on Na^+^ dynamics in the AIS (Fleidervish et al., 2010). Action potentials in the mitral cell model have the largest duration (Figure 1B, inset, blue), followed by the spikes generated by the pyramidal cell (green) and the Purkinje cell (red). Furthermore, the diffusion of Na^+^ out of the AIS in the mitral cell model is the slowest among the three. As a consequence, AIS [Na^+^]_i_ in the mitral cell model is increased during the spike train by 40 mM, as compared to the pyramidal cell (18 mM) and Purkinje cell (6 mM) models. Moreover, contrary to the pyramidal and Purkinje cell models, in the mitral cell model Na^+^ concentration does not reach equilibrium during the spike train, and thus longer trains would result in even larger [Na^+^]_i_ build-up.

The large volume of the soma prevents extreme changes in somatic [Na^+^]_i_ (Figure 1C, note the scale). The lack of good diffusion sinks, however, results in a slow return to baseline [Na^+^]_i_, governed mostly by active Na^+^ extrusion. Among the models tested, the pyramidal cell model has the highest density of voltage-gated Na^+^ channels in its soma, leading to the largest somatic [Na^+^]_i_ elevation (6 mM) of all. The only major somatic diffusion sink is the proximal dendrite, where [Na^+^]_i_ follows a similar trajectory as the soma (Figure 1D).

Similarly to the soma, no strong diffusion sinks are available near the distal dendrite (Figure 1E), hence Na^+^ concentration dynamics are determined by the density of active Na^+^ conductance and extrusion mechanisms. In the pyramidal and Purkinje cell models the level of dendritic voltage-gated Na^+^ conductance is very low, resulting in nearly no [Na^+^]_i_ build-up in the distal dendrite. Conversely, the mitral cell model has a high level of dendritic voltage-gated Na^+^ conductance (Ma and Lowe, 2004), causing a modest (4 mM) elevation in dendritic [Na^+^]_i_. Nevertheless, this elevation is extremely prolonged, since Na^+^ extrusion from the distal dendrites is governed solely by pumping, as the lack of sinks prevents effective diffusion. Such prolonged dendritic elevation in [Na^+^]_i_ has been indeed observed experimentally (Fleidervish et al., 2010; Zylbertal et al., 2015) and can potentially act as a non-leaky integrator of neuronal activity (see below).

The Purkinje cell model consists of a detailed axon with myelinated sections and nodes of Ranvier, enabling the simulation of [Na^+^]_i_ build-up along the axon. Due to the strong diffusion sinks created by the nearby myelinated sections and the limited Na^+^ conductance, [Na^+^]_i_ build-up in the third node of Ranvier (similarly to the other nodes) is relatively small and transient (Figure 1F).

Overall, a large variety of temporal profiles of [Na^+^]_i_ were observed in the different cell models as well as at different compartments of the same model. The differences among the temporal profiles reflect differences in Na^+^ conductance, Na^+^ extrusion mechanisms, and geometrical relations between neighboring compartments.

### Synaptic activity results in prolonged local elevation in [Na^+^]_i_

**Figure 2:**
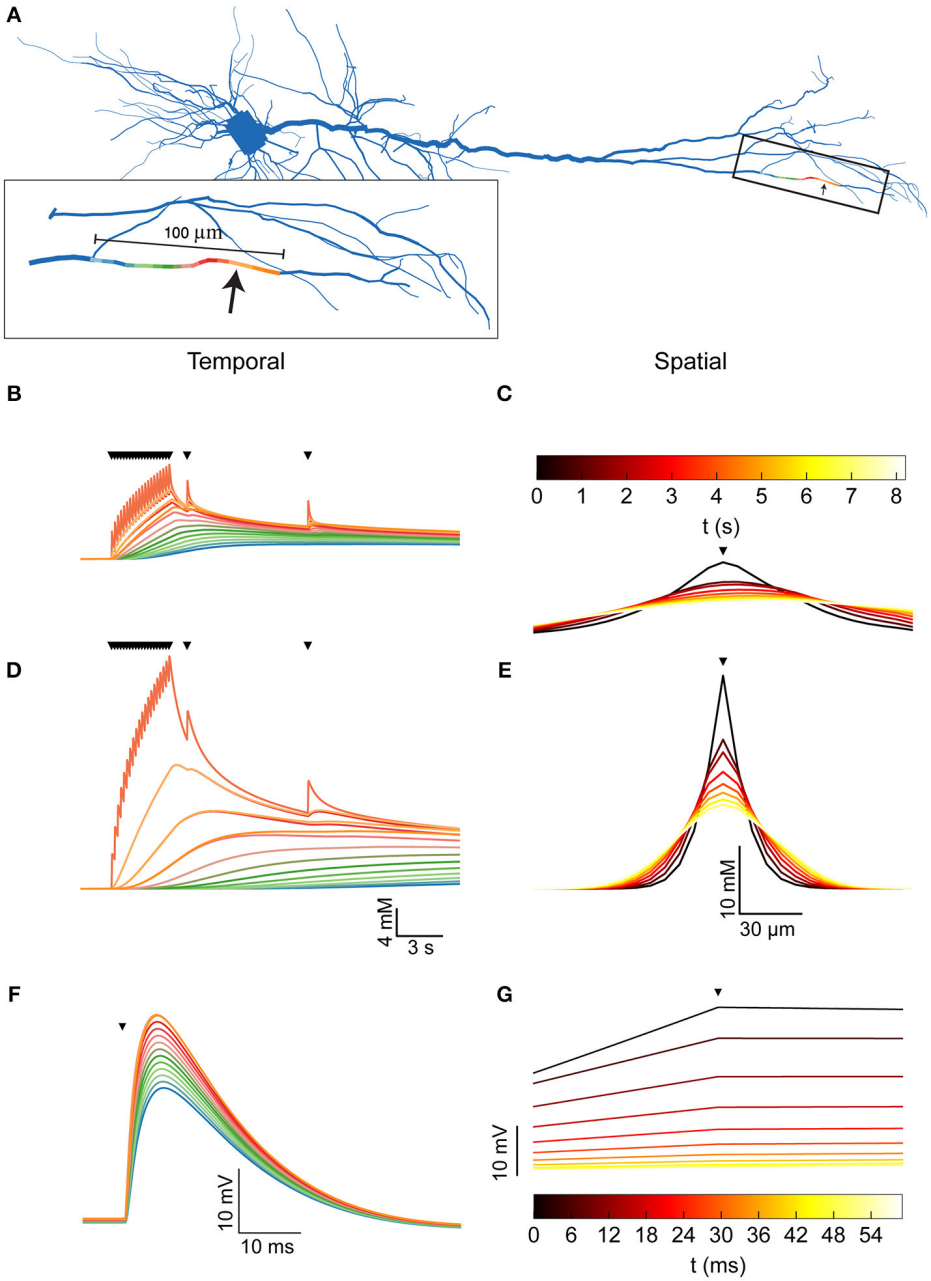
Dendritic [Na^+^]_i_ dynamics in the pyramidal model cell following a train of synaptic inputs. **(A)** The morphology of the pyramidal cell model, indicating the distal site used for simulating synaptic inputs (arrow). Inset shows a magnification of the stimulated dendrite, showing the colors used to indicate the dendritic segments below in **(B,D,F)**. **(B)** [Na^+^]_i_ dynamics in the dendritic segments color-coded in **(A)**, during and after repeated synaptic stimulations (black triangles). **(C)** The spatial profile (along the dendrite) of [Na^+^]_i_ level at different points in time following the stimulation (indicated by the color bar). The stimulation location is indicated by a black triangle. **(D,E)** similar to **(B,C)**, with a 10-fold reduction in the Na^+^ diffusion coefficient to approximate the effect of dendritic spines. **(F)** Membrane potential change (EPSP) in the dendritic segments color coded in **(A)**, due to the first synaptic stimulation (black triangle). **(G)** The spatial profile (along the dendrite) of the membrane potential change at different points in time following the peak of the EPSP (indicated by the color bar). Stimulation location is indicated by a black triangle.

Since the pyramidal and Purkinje cell models lack significant dendritic voltage-dependent Na^+^ conductance, firing activity does not alter their dendritic [Na^+^]_i_ (Figure 1E, red and green lines). We therefore examined the effect of another possible source of intracellular Na^+^, the synaptic current, on dendritic [Na^+^]_i_. Since the local dendritic properties in the context of Na^+^ are similar in the two models, we used the pyramidal cell model (Figure 2A) to demonstrate this effect.

A 5 Hz, 4-s long train of synaptic inputs, followed by two isolated subsequent inputs given to examine prolonged influences (Figure 2B, triangles) were simulated at a distal site in the apical dendritic tree (Figures 2A,B, arrow). The Na^+^ component of the synaptic conductance was inferred from the synaptic reversal potential (+10 mV), assuming that only Na^+^ and K^+^ conductances contribute to the total synaptic current. As apparent by both the temporal (Figure 2B) and the spatial (Figure 2C) profiles of the dendritic [Na^+^]_i_, the synaptic activation caused a moderate (8 mM) increase in [Na^+^]_i_ at the synaptic site, which decayed by diffusion to neighboring segments (color coded by proximity as in A) in a few seconds.

The presence of dendritic spines results in non-linear (“anomalous”) dendritic diffusion (Santamaria et al., 2011). To linearly approximate the effect of dendritic spines, which drastically reduce Na^+^ spread (resulting in a smaller apparent Na^+^ diffusion coefficient), we repeated the simulation with a 10-fold reduction in the dendritic Na^+^ diffusion coefficient. Compared to a spiny dendrite, this reduction results in a conservative estimation of [Na^+^]_i_ in the stimulated site and a good approximation of [Na^+^]_i_ in neighboring sites, as demonstrated by simulating the two cases (Supplementary Figure). Indeed, under these conditions (Figures 2D,E), the change in [Na^+^]_i_ is much larger (25 mM), more localized, and persists for a longer period of time.

Compared with the changes in dendritic [Na^+^]_i_, alterations in membrane potential due to synaptic activity (Figures 2F,G) are considerably faster and have a larger spatial extent. Synapse-driven [Na^+^]_i_ changes are therefore more compartmentalized than electrical changes and persist for longer periods of time.

### Elevated [Na^+^]_i_ causes a reduction in Na^+^ nernst potential, affecting the amplitude of action potentials and EPSPs

**Figure 3:**
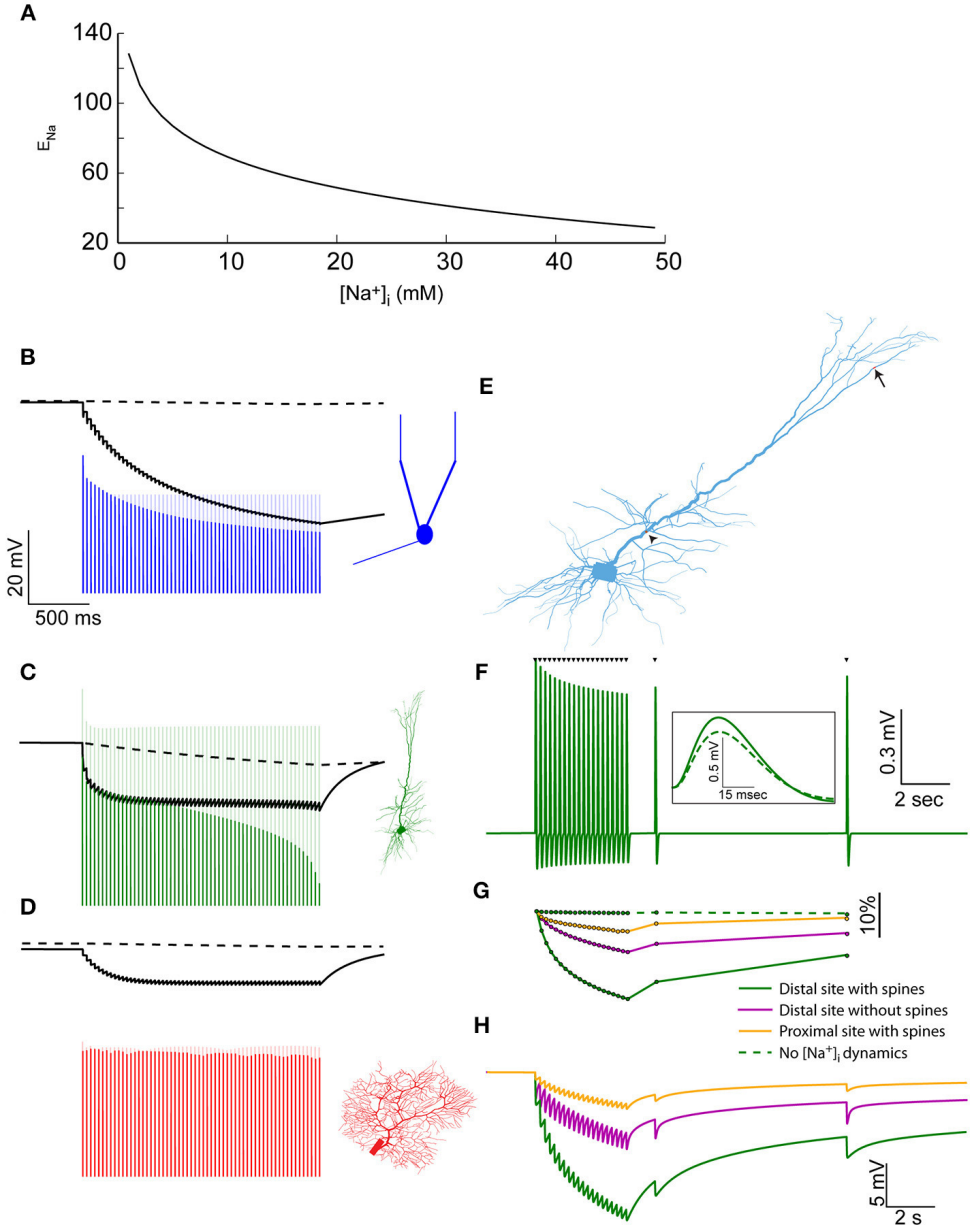
Elevated [Na^+^]_i_ causes a reduction in Na^+^ Nernst potential, affecting the amplitude of action potentials and EPSPs. **(A)** The relationship between [Na^+^]_i_ and E_Na_, assuming physiological temperature and [Na^+^]_o_ = 150 mM. **(B)** The modulation of spike amplitude during a 30 Hz train in the mitral cell model (blue) is due to the change in AIS E_Na_ (black line), and not in somatic E_Na_ (dashed black line). A model without Na^+^ accumulation shows no such spike amplitude adaptation (light blue line). The voltage trace is truncated to show only the peaks of the spikes. **(C)** Similar simulation using the pyramidal cell model, where similar amplitude adaptation is caused by both AIS and somatic changes in E_Na_. **(D)** In the Purkinje cell model no spike amplitude adaptation is observed. **(E)** Indicating the distal synaptic stimulation site (arrow) and the proximal one (arrowhead). **(F)** Somatic recording of EPSPs generated by a repeated stimulation of the distal site (when approximating the effect of dendritic spines). Inset – a magnification showing the first (solid) and last (dashed) EPSP from the train. **(G)** The changes in normalized EPSP amplitude in the simulation presented in **(F)** (solid green line), without approximating the effect of dendritic spines (magenta line), when stimulating the proximal site (orange line) and without accounting for [Na^+^]_i_ changes (dashed green line). **(H)** The changes in E_Na_ at the site of synaptic stimulation when using the distal site with (solid green line), or without (magenta line) approximating the effect of dendritic spines, or using the proximal site (orange line).

One potential outcome of an increase in [Na^+^]_i_ is a reduction in the Na^+^ Nernst potential (E_Na_). The relationship between [Na^+^]_i_ and E_Na_, assuming physiological temperature and [Na^+^]_o_ = 150 mM, is shown in Figure 3A. As apparent, starting from the physiological levels of baseline [Na^+^]_i_ (about 10–20 mM), each 10 mM increase in [Na^+^]_i_ results in a reduction in E_Na_ of about15 mV. Therefore, an increase in [Na^+^]_i_ due to firing (Figure 1) or synaptic (Figure 2) activity should significantly change E_Na_. Naturally, such changes would affect the membrane potential only during episodes where the Na^+^ conductance is large, such as the peaks of action potentials and EPSPs.

To assess these effects, we first examined the somatic spike amplitudes during the 30 Hz train evoked by current pulses. In the AOB mitral cell (Figure 3B), a considerable reduction in spike amplitude is apparent (blue line) compared to a model that does not take [Na^+^]_i_ changes into account (light blue line). Such amplitude adaptation is widely observed experimentally (Le Bé et al., 2007), and could not be reproduced using simple Hodgkin-Huxley dynamics (Zylbertal et al., 2015). It is explained by a reduction in E_Na_ at the AIS (Figure 3B, solid black line), rather than the somatic E_Na_ (dashed line), which does not change along the train. Recovery from this amplitude reduction would follow the [Na^+^]_i_ profile in the AIS (Figure 1B), meaning that subsequent action potentials generated during several seconds following the train would also have reduced amplitude. It should be noted that a change in E_Na_ is only one of many possible underlying causes for spike amplitude adaptation, and experimental work will be needed to assess its relative contribution to the phenomenon.

A similar effect is observed when simulating the spike train in the pyramidal cell (Figure 3C), where the amplitude adaptation stems from changes in both the somatic and AIS E_Na_. It should be noted that the original pyramidal cell model diverged from the experimental results by not reproducing a spike amplitude adaptation during a test pulse (Hay et al., 2011). As shown here, introducing Na^+^ dynamics to this model bridges this gap between the model and the experimental results.

In the Purkinje cell model (Figure 3D), little change in the somatic or AIS E_Na_ is inferred from [Na^+^]_i_, and the peaks of the fast action potentials are far below E_Na_, causing a near-constant spike amplitude, as previously observed experimentally (Llinás and Sugimori, 1980).

In order to examine the effect of E_Na_ changes on synaptic inputs, we reverted to the synaptic stimulation pattern we described above in the pyramidal cell model (Figure 2), this time using both distal and proximal dendritic sites (Figure 3E, arrow and arrowhead). Figure 3F presents the EPSPs recorded in the soma in the case of a distal stimulation site, when a low Na^+^ diffusion constant was used (approximating dendritic spines). As apparent, the EPSP amplitudes are reduced along the input train, in a manner that resembles weak short-term synaptic depression. Subsequent inputs, at 2 and 9 s after the train, demonstrate the slow recovery of the EPSP amplitude.

The normalized somatic EPSP amplitude (Figure 3G, green line) shows a 17% reduction in amplitude by the end of the train. A smaller reduction was found when the effect of spines was not taken into account (magenta line) or when the proximal stimulation site, which has a larger dendritic diameter, was examined (orange line). In a model without Na^+^ dynamics, no EPSP amplitude reduction is observed (dashed green line).

As in the case of action potentials, the EPSP amplitude reduction is explained by a local change in E_Na_. This change is maximal in the distal stimulation site when the effect of dendritic spines was included (Figure 3H, green line), intermediate when the effect of spines was excluded (magenta line), or small when the synaptic input was located at a proximal segment (orange line).

In conclusion, changes in [Na^+^]_i_ result in significant and possibly prolonged changes in both action potential and EPSP amplitude, especially following bouts of activity. Changes in action potential amplitude may not propagate down the axon, therefore, the degree of their functional significance is not clear. Changes in EPSP amplitude are likely to influence neuronal information processing, reminiscent of synaptic depression.

### Elevated [Na^+^]_i_ causes an increase in Na^+^-K^+^ pump outward current, which may result in spike frequency adaptation and prolonged after-hyperpolarization

**Figure 4:**
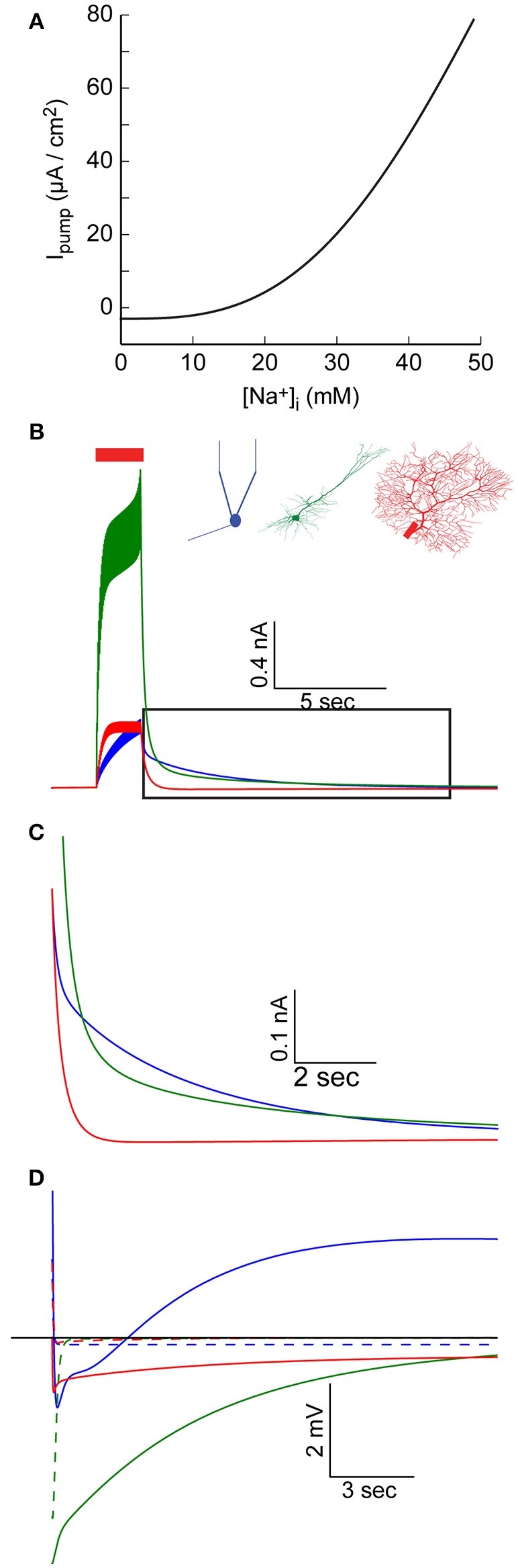
Elevated [Na^+^]_i_ causes an increase in Na^+^-K^+^ pump outward current. **(A)** The relationship between [Na^+^]_i_ and the specific Na^+^-K^+^ pump current, using pump parameter fitted for the AOB mitral cell model. **(B)** AIS Na^+^-K^+^ pump current during and after a 30 Hz spike train in the AOB mitral cell model (blue), the pyramidal cell model (green), and Purkinje cell model (red). **(C)** Magnification of the period following the train (indicated by a box in **B**), showing prolonged inward current in the mitral and pyramidal cells. **(D)** Membrane potential in the three cells following the train, compared to simulations without [Na^+^]_i_ changes (dashed lines).

Similarly to any enzyme, the electrogenic Na^+^-K^+^ pump rate depends upon the concentration of its substrates, [Na^+^]_i_ and [K^+^]_o_. Since our models assume constant [K^+^]_o_, we will demonstrate the effect of changing [Na^+^]_i_ on the outward current generated by the pump. The pump properties (forward and reverse time constants) were taken from the fitted AOB mitral cell model. Figure 4A shows the relationship between [Na^+^]_i_ and the pump current per cm^2^ (assuming the pump density is equal to the one used in the axon hillock of the mitral cell). Large changes in the pump current are expected, especially when the [Na^+^]_i_ level is above 20 mM. The pump can therefore be thought of as a Na^+^-dependent outward current mechanism, similar to the Slick and Slack Na^+^-dependent K^+^ channels (Kaczmarek, 2013). Like these mechanisms, it may act as a homeostatic mechanism by preventing runaway activity.

Such variations in the pump current can have significant effects, particularly when large changes in [Na^+^]_i_, occur. We therefore examined the current generated by the AIS pumps during and after an evoked train of action potentials (Figure 4B). This current is a function of both AIS [Na^+^]_i_ and the density of Na^+^-K^+^ pumps in the AIS, hence it does not perfectly mirror the AIS [Na^+^]_i_ build-up (Figure 1B). In the AOB mitral cell model, this current keeps intensifying during the train, and reaches a peak value of 400 pA (Figure 4B, blue), while in the Purkinje cell model, a steady-state current with a similar amplitude is quickly established (Figure 4B, red). The strongest current is observed in the pyramidal cell model (Figure 4B, green) due to the combination of high [Na^+^]_i_ and a high density of pumps.

During a prolonged firing burst, such a current may cause spike frequency adaptation (not evident here, since each spike is evoked by an individual current pulse), but its effect may be masked by multiple conductances, which are active during a firing episode (such as voltage- and Ca^2+^-dependent K^+^ conductances). Yet, due to the relatively slow Na^+^ dynamics, the elevated Na^+^-K^+^ pump-mediated outward current may persist for a long period following the burst (Figure 4C), a time in which most active conductances return to their resting state. This effect is especially evident in the mitral cell and pyramidal cell models (blue and green lines), whereas in the Purkinje cell model, the somatic sink results in rapid restoration of AIS [Na^+^]_i_ (red line, Figure 1B) and a fast reduction in the pump current.

The immediate result of this modulation of the Na^+^-K^+^ current is a prolonged hyperpolarizing potential that follows each burst of action potentials (Figure 4D, solid lines, vs. dashed lines representing results without modeling Na^+^ mechanisms). Notably, the initial part of such hyperpolarization may be related to Ca^2+^-dependent K^+^ conductance. In the case of the mitral cell model (blue line), the latter part of the response is masked by a prolonged Ca^2+^-dependent inward current. The interplay between these two currents underlies the generation of infra-slow oscillations (Cressman et al., 2009; Zylbertal et al., 2017), where the Na^+^-K^+^ pump current acts as an adaptation current that terminates each firing burst and determines the inter-burst interval duration.

### The coupling between Na^+^ and Ca^2+^ dynamics leads to prolonged Ca^2+^ transients that may tag active synapses

**Figure 5:**
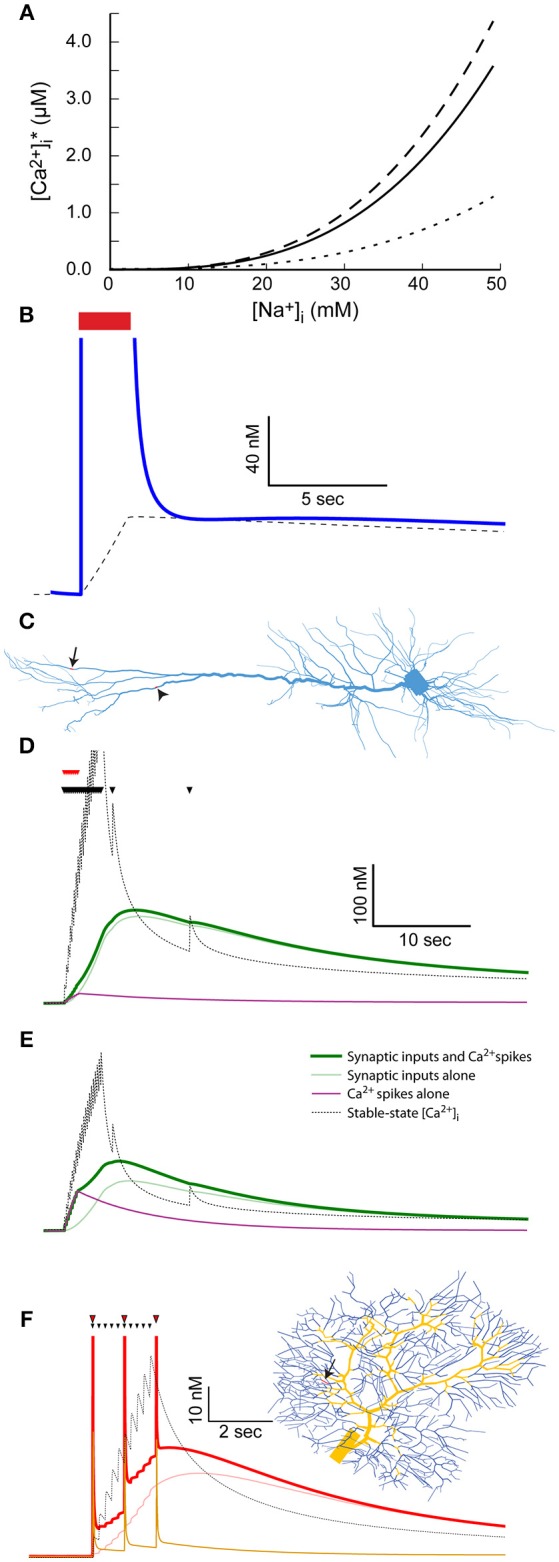
The coupling between Na^+^ and Ca^2+^ dynamics leads to prolonged Ca^2+^ transients and labeling of active synapses. **(A)** The relationship between [Na^+^]_i_ and stable-state [Ca^2+^]_i_ calculated using the parameters used for the mitral cell dendritic tuft (solid line) or a 10-fold reduction (dotted line) or increase (dashed line) in the Na^+^-Ca^2+^ exchanger density. **(B)** The stable-state [Ca^2+^]_i_ (dashed black line) and actual [Ca^2+^]_i_ (blue line) in the dendritic tuft of the mitral cell model during and after a 30 Hz spike train (red bar). **(C)** Indicating the distal dendritic site (arrow) and “hot zone” site (arrowhead) used to test the effect of synaptic stimulation on [Ca^2+^]_i_ dynamics in the pyramidal cell model. **(D)** The effect of repeated synaptic stimulation (black triangles) in the distal site on local [Ca^2+^]_i_ with (thick green line) or without (light green line) concurrent back-propagating Ca^2+^ spikes (red triangles). The effect of the calcium spikes alone is indicated by a magenta line, and the stable-state [Ca^2+^]_i_ by a black dotted line. **(E)** Similar to **(C)**, except for stimulation in the Ca^2+^ “hot zone” (the site indicated by an arrowhead in **C**). **(F)** The effect of repeated parallel fiber stimulation (black triangles) on local [Ca^2+^]_i_ with (thick red line) or without (light red line) concurrent climbing fiber stimulation (red triangles). The parallel fiber stimulation site is indicated by an arrow on the dendritic tree (right) and the climbing fiber input sites by yellow segments. The effect of the complex spikes alone is indicated by an orange line, and the stable-state [Ca^2+^]_i_ by a black dotted one.

Another interesting consequence of the changes in [Na^+^]_i_ is their influence on Ca^2+^ extrusion, particularly via the Na^+^-Ca^2+^ exchanger. This is the main mechanism for removal of a large excess of Ca^2+^, such as the one that develops during a burst of spikes by the activity of voltage-activated Ca^2+^ channels (Fierro et al., 1998). Contrary to the Ca^2+^ pump, the Na^+^-Ca^2+^ exchanger does not rely on ATP hydrolysis for Ca^2+^ extrusion but rather uses the trans-membrane Na^+^ gradient to remove calcium from the cell. As a consequence, elevation in [Na^+^]_i_ leads to reduction in the rate of Ca^2+^ extrusion by the exchanger, and sometimes even to calcium influx (Schäfer, 2001; Jeon et al., 2003). Assuming that the Na^+^-Ca^2+^ exchanger and the Ca^2+^ pump are the only mechanisms that transfer Ca^2+^ across the membrane (i.e., when voltage-gated calcium channels are closed), [Ca^2+^]_i_ stable-state is a dynamic equilibrium, where Ca^2+^ flows out through the pump and in through the exchanger. The level of this stable-state [Ca^2+^]_i_ is therefore a function of [Na^+^]_i_ that may be inferred analytically (see Methods) when treating the latter as fixed (an assumption justified by its slow dynamics compared to [Ca^2+^]_*i*_). This function is plotted in Figure 5A, with the pump and exchanger parameters fitted for the mitral cell model (solid line), or in cases of a 10-fold increase (dashed line) or decrease (dotted line) in density of the Na^+^-Ca^2+^ exchanger. The [Ca^2+^]_i_ stable-state is thus determined by the exchanger to pump ratio: a larger ratio results in stronger influence of [Na^+^]_i_.

Since [Na^+^]_i_ in the distal dendrite of the mitral cell model remains elevated for a long period of time following a spike train (Figure 1E), so does the dendritic stable-state [Ca^2+^]_i_ (Figure 5B, dashed line). Following the spike train, the dendritic [Ca^2+^]_i_ quickly drops to the vicinity of the stable-state [Ca^2+^]_i_, and subsequently follows its slow decay (Figure 5B, blue line). This results in a prolonged [Ca^2+^]_i_ elevation that, in the case of the AOB mitral cell that expresses Ca^2+^-dependent non-selective cation channels, may cause a prolonged inward current underlying persistent activity (Zylbertal et al., 2015).

In the pyramidal and Purkinje cell models, the dendritic active Na^+^ conductance is too low to account for a similar effect. Synaptic activity may, however, produce comparable, albeit local, elevation in [Na^+^]_i_ (Figure 2). We therefore tested the result of synaptic input on [Ca^2+^]_i_ dynamics in two input sites: one is the distal site in the dendritic tuft (Figure 5C, arrow), and the other is a distal site located within the Ca^2+^ “hot zone” of the model (700 μm from the soma, Figure 5C, arrowhead). As before, the stimulation pattern included a 4-s, 5 Hz train of inputs and two subsequent isolated inputs (Figure 5D, black triangles), using the low Na^+^ diffusion coefficient to approximate the effect of dendritic spines.

Stimulating in the original distal site (Figure 5D) results in a prolonged [Ca^2+^]_i_ elevation (120 nM peak, light green line) generated by the change in local stable-state [Ca^2+^] (black dotted line). Note that the density of Ca^2+^ pump and Na^+^-Ca^2+^ exchanger is too low for the actual [Ca^2+^]_i_ to closely follow the stable-state [Ca^2+^]_i_ during the train of inputs (i.e., the light green line is initially below the dotted black line). Later, when local [Na^+^]_i_ slowly decays and so does the stable-state [Ca^2+^]_i_, the actual [Ca^2+^]_i_ closely follows. Thus, the active synapses elicited a prolonged local increase in Ca^2+^ concentration that may lead to local tagging of the active synapse (Martin and Kosik, 2002). It should be noted that such a tagging mechanism operates without activation of Ca^2+^ conductance.

To examine the effect of additional Ca^2+^ influx through voltage-gated channels, we used somatic current injections concurrent with the initial synaptic inputs to simulate eight calcium spikes, activated by back-propagating action potentials (Figure 5D, red triangles, Larkum et al., 2001; Hay et al., 2011). Since the density of Ca^2+^ channels in the examined compartment is low, the calcium spikes did not significantly alter the [Ca^2+^]_i_ trajectory (Figure 5D, thick green line). The effect of the calcium spikes alone, without synaptic inputs, on [Ca^2+^]_i_ is shown by the magenta line.

In contrast, when repeating a similar simulation in the “hot zone” site (Figure 5E), the calcium spikes evoked by somatic current injection had a significant effect on the [Ca^2+^]_i_ trajectory (thick green line vs. light green line, peaks at 110 and 75 nM, effect of calcium spikes alone shown by magenta line). Notably, since the actual [Ca^2+^]_i_ is below stable-point [Ca^2+^]_i_ during the train of inputs, additional [Ca^2+^]_i_ influx at this time contributes to the elevation in [Ca^2+^]_i_ for a prolonged time. In this case, the system acts as a coincidence detector, where back-propagating spikes, concurrent with the train of synaptic inputs, result in stronger Ca^2+^ tagging of the active synapses.

A similar effect was observed in a Purkinje cell simulation, where climbing fiber inputs, evoking complex spikes that are associated with global [Ca^+^]_i_ influx, were used instead of back-propagating Ca^2+^ spikes. A 2-s 5 Hz train of parallel fiber inputs were simulated in a single site of the dendritic tree (Figure 5F, black triangles, location indicated by arrowhead). As in the case of the pyramidal cell, the train of synaptic stimulation alone resulted in a prolonged increase in local [Ca^2+^]_i_ (Figure 5F, light red line), which initially remained below the stable-state [Ca^2+^]_i_, and later followed it (Figure 5F, black dotted line). Climbing fiber stimulation was simulated by stimulating all segments of the dendritic tree thicker than 2.2 μm (Figure 5F, yellow segments of the dendritic tree). These stimulations were given at 1 Hz, concurrent with the parallel fiber input train (red triangles). The contribution of Ca^2+^ influx due to the ensuing complex spikes caused a larger overall [Ca^2+^]_i_ elevation (52 nM vs. 45 nM peak), a difference that persisted for several seconds following the inputs. The effect of climbing fiber stimulation alone is shown using an orange line. Again, this case represents coincidence detection, where active synapses are more strongly tagged by Ca^2+^ in case of simultaneous climbing and parallel fiber inputs.

In conclusion, the coupling of Na^+^ and Ca^2+^ dynamics by the Na^+^-Ca^2+^ exchanger causes a prolonged global dendritic [Ca^2+^]_i_ elevation in the mitral cell model, and a local [Ca^2+^]_i_ elevation in the vicinity of active synapses in the Purkinje and pyramidal cell models. The latter effect could result in tagging of active synapses, intensified in some cases by concurrent back-propagating calcium spikes (in the pyramidal cell model) or complex spikes (in the Purkinje cell model).

### The described effects on neuronal computation are preserved under realistic distributed input

To test the validity of the effects shown above under a more realistic setting, we simulated the pyramidal cell under a complex set of inputs mimicking realistic cortico-cortical and thalamo-cortical connections. This was done by simulating 1,000 pre-synaptic “cortical” spike trains that follow a Poisson distribution (average firing rate of 1 Hz). Each train stimulated five excitatory connections uniformly distributed along the dendritic tree. Additionally, we employed 1,000 pre-synaptic “thalamic” spike trains. These were set to background average firing rate of 1 Hz with three epochs representing sensory stimulus, where the average firing rate was elevated to 5 Hz. The thalamic connections were clustered (all five connections were co-localized), and were more densely distributed in proximal dendrites (see Methods).

**Figure 6:**
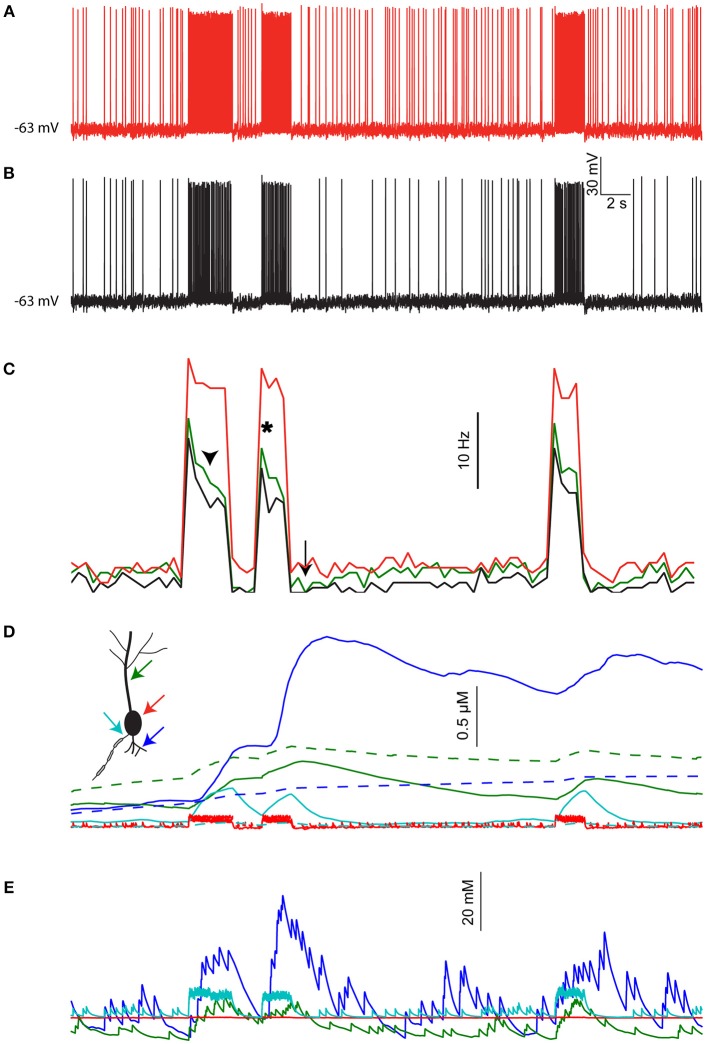
The effects of [Na^+^]_i_ dynamics are preserved under realistic distributed input. **(A)** Somatic membrane potential in the pyramidal cell model driven by simulated local inputs and clustered thalamic sensory evoked inputs (red bars), where [Na^+^]_i_ is kept fixed throughout the simulation. **(B)** Somatic membrane potential in the same model using identical inputs but allowing [Na^+^]_i_ to change. **(C)** Average firing rate histogram (*n* = 3 runs) without [Na^+^]_i_ changes (black), with [Na^+^]_i_ changes (red), and when using nonclustered thalamic inputs without accounting for dendritic spines (green). **(D)** [Ca^2+^]_i_ calculated in the simulation shown in **(B)** for a proximal basal dendrite (blue), apical dendrite (green), soma (red), and AIS (cyan). Dashed lines show [Ca^2+^]_i_ dynamics when [Na^+^]_i_ is held fixed. **(E)** [Na^+^]_i_ in the same compartments shown in **(D)**.

Figure 6A shows the resulting activity when not taking changes in [Na^+^]_i_ into account (i.e., conventional model behavior). The cell exhibits constant background firing rate of ~7 Hz, and elevated firing rate during epochs of sensory stimuli (red bars). Introducing [Na^+^]_i_ dynamics (with a low diffusion coefficient, representing the effect of dendritic spines) under identical pre-synaptic spike trains and contact location (Figure 6B) causes several changes in the cell output. These changes are more pronounced when comparing the firing rate histogram of the two cases, averaged over three runs of the simulation (Figure 6C, red vs. black lines). First, the average background firing rate is lowered due to dendritic Na^+^ accumulation that reduces the synaptic reversal potential. More importantly, epochs of elevated firing rate cause a subsequent prolonged reduction in background firing rate (arrow), as well as in sensory evoked firing rate during the course of the same epoch (arrowhead) and subsequent epochs (asterisk). These effects are slightly reduced in a simulation where the local [Na^+^]_i_ changes are lower, for example, when the thalamic inputs are not clustered and the diffusion coefficient is not adjusted to account for dendritic spines (Figure 6C, green line).

Another consequence of [Na^+^]_i_ dynamics is integration of synaptic activity by the local Ca^2+^ level. This is shown in Figure 6D for a basal dendrite with high density of thalamic contacts (Figure 6D, blue line) and an apical dendrite with less thalamic inputs (green line). Somatic and AIS [Ca^2+^]_i_ changes are smaller and transient (Figure 6D, red and cyan lines respectively). For comparison, [Ca^2+^]_i_ dynamics under fixed [Na^+^]_i_ conditions are shown with dashed lines. The [Na^+^]_i_ dynamics underlying these effects are shown for the same four compartments in Figure 6E. Thus, segments having strong synaptic inputs are more effectively tagged by [Ca^2+^]_i_.

To conclude, the effects of [Na^+^]_i_ dynamics demonstrated above using a “clean” stimulus (isolated spatially and temporally) are also applicable when driving the cell using stochastic and distributed inputs.

## Discussion

In this study, we used three realistic neuronal models to explore the predicted dynamics of [Na^+^]_i_ in various cellular compartments and the possible effects these dynamics might have on neuronal computation. Our results show that [Na^+^]_i_ can affect neuronal information processing by changes in E_Na_, the Na^+^-K^+^ pump current, and the Na^+^-Ca^2+^ exchanger rate. The main effects we encountered are spike amplitude adaptation, apparent synaptic depression, prolonged after-hyperpolarization, and prolonged and localized elevation in [Ca^2+^]_i_, which can be used as a tagging mechanism of active synapses.

### Functional significance of the sodium accumulation

The effects we describe may have several possible implications on neuronal computation:

#### EPSP amplitude modulation

We show that repeated activation of an individual synapse results in gradual and sustained reduction of the EPSP amplitude due to local Na^+^ accumulation and E_Na_ reduction (Figures 3E–H). This effect is similar to the effect of synaptic saturation (Bush and Sejnowski, 1994), only acting at much longer time scales. It can be seen as a post-synaptic mechanism of use-dependent synaptic depression, which operates primarily at distal synaptic locations, and reduces the impact of redundant vs. novel inputs. A similar effect was observed when action potential amplitude was measured, but its implication on neuronal computation is not clear. In some cases, however, it might be used as an experimental proxy to measure AIS and/or somatic [Na^+^]_i_ changes.

#### Prolonged activity-dependent hyperpolarization

Na^+^ accumulation during activity results in increased activity of the Na^+^-K^+^ pump, leading to hyperpolarization of the membrane potential. Since the bulk of this current is in the AIS, its effect is mainly on the output of the neuron. This outcome may protect neurons from runaway positive feedback by limiting the duration of action potential bursts, and acting as a short-term memory of past spiking activity (Pulver and Griffith, 2010; Zhang and Sillar, 2012).

#### Prolonged dendritic Ca^2+^ elevation

According to our model, the coupling of Na^+^ and Ca^2+^ dynamics will result in prolonged local Ca^2+^ elevations whenever activity evokes a prolonged Na^+^ elevation (Figure 5). This will happen in cases of sufficient density of dendritic voltage-gated Na^+^ channels (as in the AOB mitral cell, Figure 5B), or due to repeated synaptic stimulation (Figures 5C–F). The latter is a possible mechanism for specific tagging of active synapses, where [Ca^2+^]_i_ elevation could activate cellular pathways related to gene expression and synaptic plasticity (Martin and Kosik, 2002). Such tagging would be gradual, since repeated synaptic activation will result in a further increase in [Ca^2+^]_i_, due to the wide dynamic range of [Na^+^]_i_. Since Ca^2+^ is an important second messenger, prolonged elevation in its concentration could have several other end results. For example, it could directly gate a K^+^ or non-specific ion currents, thus causing prolonged membrane potential changes.

Our results further show that, in some cases, the amplitude of the long-lasting post-stimulus [Ca^2+^]_i_ reflects the coincidence of specific synaptic activity and backpropagating action potentials or complex spikes. In the pyramidal cell model, this effect occurs in synapses located near the Ca^2+^ conductance hot-zone (700 μm from the soma in the apical dendrite), triggered by a short train of Ca^2+^ spikes evoked by backpropagating action potentials. A similar effect was shown for the concurrent activation of parallel fiber synapses and the climbing fiber synapse in Purkinje cells, triggered by the global [Ca^2+^]_i_ influx caused by a complex spike. This effect may contribute to spike timing-dependent plasticity over extended time-scales, since it is evident only when backpropagating or complex spikes follow the synaptic inputs.

The common trait among these possible outcomes is the long time-scale, resulting from the relatively slow Na^+^ dynamics. This reflects the potential role of Na^+^ as a slow chemical integrator of past activity, as intense bouts of activity are needed to effectively change its level, yet its decay to baseline is relatively slow. This contrasts with Ca^2+^, the concentration of which rapidly rises as a function of neuronal activity, and rapidly declines as Ca^2+^ is buffered, stored, and actively extruded from the cytoplasm.

### Sensitivity to chosen parameters and model design

Since our goal in this study was to explore the possible effects of changes in [Na^+^]_i_ rather than to accurately reproduce experimental findings, some of the parameter values we chose for the Na^+^-related mechanisms were not highly constrained. In particular, we often applied values fitted using experimental results in the AOB mitral cell model to the pyramidal and Purkinje cell models and had to guess the densities of pumps and exchangers in several compartments (see Methods). Some aspects of the original models (e.g., the axonal structure and the distribution of several conductances) were themselves poorly constrained, increasing the uncertainty in the revised models. Most of our conclusions, however, hold with varying levels of confidence despite these uncertainties.

First, most of our conclusions regarding the dynamics of [Na^+^]_i_ themselves (Figures 1–2) are well constrained, since they rely on the density of voltage-gated or synaptic Na^+^ conductance, cellular morphology, and diffusion coefficient—properties which are adequately constrained (except for the way dendritic spines should affect the apparent diffusion coefficient—hence we present simulation results with and without this correction). The agreement between the SERs calculated using the models and the ones experimentally measured validates the active Na^+^ current dynamics used in the models. Therefore, conclusions regarding changes in E_Na_ and their effects (Figure 3) are also constrained as a direct consequence of the morphology and the synaptic or voltage-gated current.

Regarding Na^+^-K^+^ pumps, Ca^2+^ pumps, and Na^+^-Ca^2+^ exchangers (Figures 4–5), the specific profile of their effects indeed depends on parameters such as ionic affinities and mechanism densities. Nevertheless, their outcome should at least resemble the one we describe, as long as two assumptions regarding Na^+^ and Ca^2+^ extrusion in neurons are accepted: (a) The bulk of Na^+^ extrusion is carried out by the electrogenic Na^+^-K^+^ pump, which is less significant for the [Na^+^]_i_ dynamics than diffusion, at least for short time scales (Fleidervish et al., 2010). (b) The bulk of Ca^2+^ extrusion (particularly in cases of large Ca^2+^ excess) is carried out by the Na^+^-Ca^2+^ exchanger (Fierro et al., 1998). These assumptions, combined with our results concerning [Na^+^]_i_ dynamics, necessarily lead to conclusions similar to ours.

### Experimental findings

This modeling work was inspired by our experimental findings in AOB mitral cells, where we observed several of the effects discussed above by measuring [Na^+^]_i_, [Ca^2+^]_i_, and membrane potential (Zylbertal et al., 2015). We demonstrated the involvement of the Na^+^-K^+^ pump current in the responses of these cells by observing its voltage independence and its sensitivity to ouabain (Na^+^-K^+^ pump blocker) and replacement of Na^+^ with Li^+^. The effect mediated by the Na^+^-Ca^2+^ exchanger was also inferred using Na^+^ replacement by Li^+^, which does not bind to the exchanger. Moreover, [Na^+^]_i_ dynamics were directly measured using Na^+^ imaging (SBFI, Rose et al., 1999).

Rose and Konnerth (2001) observed strong and prolonged Na^+^ transients in dendrites and spines of hippocampal pyramidal cells following synaptic stimulation, which were qualitatively similar to the results of our simulation (Figure 2). In their case, NMDA receptors mediated the bulk of Na^+^ influx, hence, contrary to our model, it was only observed when suprathreshold stimulation was applied. As in our model, dendritic voltage-gated Na^+^ conductance hardly contributed to the observed Na^+^ accumulation.

Forrest et al. (2012) used experimental and computational methods to demonstrate the involvement of the Na^+^-K^+^ pump current in shaping the firing patterns of Purkinje cells, including prolonged after-hyperpolarization. Scheuss et al. (2006) showed how activity-dependent [Na^+^]_i_ elevation modulates Ca^2+^ extrusion by the Na^+^-Ca^2+^ exchanger in hippocampal pyramidal cells, and Kiedrowski et al. (1994) demonstrated that glutamate-evoked Na^+^ elevations impair Ca^2+^ extrusion in cerebellar granule cells.

Eilers et al. (1995) observed AMPA receptor-dependent local fast Ca^2+^ transients at sites of parallel fiber synapse activation in cerebellar Purkinje cells, but showed that the bulk of Ca^2+^ influx originates from voltage-dependent Ca^2+^ channels. Konnerth et al. (1998) applied a train stimulus to the same synapse and observed a prolonged Ca^2+^ transient caused by Ca^2+^ release from internal stores. These two observations are in disagreement with our proposed mechanism, where modulation of stable-state [Ca^2+^]_i_ (Figures 5C–F) at the site of synaptic stimulation is independent of voltage-gated Ca^2+^ conductance or release from internal Ca^2+^ stores. This discrepancy may be explained by the authors' use of a single pulse stimulation or a single short burst of pulses, producing local change in [Na^+^]_i_ that is insufficient to produce the effect we propose. A short burst of inputs may mimic the real granule cell discharge pattern in case of an isolated stimulus (Chadderton et al., 2004), but is too short to account for granule cell activity during locomotion (Powell et al., 2015).

Some of the predictions we present tend to elude experimental observation using conventional methods, possibly due to two major reasons: (a) time scale bias: these dynamics are typically longer than those usually considered in electrophysiological or imaging experiments, thus they might end up filtered out or not being noticed. (b) weak stimuli: significant effects require Na^+^ build-up by sufficiently high-frequency or prolonged stimulation, rather than a single action potential or brief synaptic input. Thus, effective experimental examination of the phenomena described by us requires strong stimuli and prolonged recording epochs.

Overall, our results demonstrate that while the assumption of constant [Na^+^]_i_ holds in short time scales and during transient or low frequency activity, changes in [Na^+^]_i_ are need to be taken into account in multicompartmental neuronal modeling when considering long time scales, synaptic plasticity, and extensive synaptic input or bursting.

## Author contributions

AZ designed and wrote the simulations and analyzed the data. AZ, YY, and SW wrote the manuscript.

### Conflict of interest statement

The authors declare that the research was conducted in the absence of any commercial or financial relationships that could be construed as a potential conflict of interest. The reviewer MS and handling Editor declared their shared affiliation.

## References

[B1] Bahl A. Stemmler M. B. Herz A. V. M. M. Roth A. (2012). Automated optimization of a reduced layer 5 pyramidal cell model based on experimental data. J. Neurosci. Methods 210, 22–34. 10.1016/j.jneumeth.2012.04.006 22524993

[B2] Barreto E. Cressman J. R. (2011). Ion concentration dynamics as a mechanism for neuronal bursting. J. Biol. Phys. 37, 361–373. 10.1007/s10867-010-9212-6 22654181 PMC3101327

[B3] Ben-Ari Y. (2002). Excitatory actions of gaba during development: the nature of the nurture. Nat. Rev. Neurosci. 3, 728–739. 10.1038/nrn920 12209121

[B4] Blaustein M. P. Lederer W. J. (1999). Sodium/Calcium exchange: its physiological implications. Physiol. Rev. 79, 763–854. 10390518 10.1152/physrev.1999.79.3.763

[B5] Bush P. C. Sejnowski T. J. (1994). Effects of inhibition and dendritic saturation in simulated neocortical pyramidal cells. J. Neurophysiol. 71, 2183–2193. 7523612 10.1152/jn.1994.71.6.2183

[B6] Carafoli E. (1991). Calcium pump of the plasma membrane. Physiol. Rev. 71, 129–153. 1986387 10.1152/physrev.1991.71.1.129

[B7] Carter B. C. Bean B. P. Forsythe I. D. Kaczmarek L. K. McCormick D. A. Welker E.. (2009). Sodium entry during action potentials of mammalian neurons: incomplete inactivation and reduced metabolic efficiency in fast-spiking neurons. Neuron 64, 898–909. 10.1016/j.neuron.2009.12.011 20064395 PMC2810867

[B8] Chadderton P. Margrie T. W. Häusser M. (2004). Integration of quanta in cerebellar granule cells during sensory processing. Nature 428, 856–860. 10.1038/nature02442 15103377

[B9] Colbert C. M. Pan E. (2002). Ion channel properties underlying axonal action potential initiation in pyramidal neurons. Nat. Neurosci. 5, 533–538. 10.1038/nn0602-857 11992119

[B10] Courtemanche M. Ramirez R. J. Nattel S. (1998). Ionic mechanisms underlying human atrial action potential properties: insights from a mathematical model. Am. J. Physiol. Hear. Circ. Physiol. 275, H301–H321. 9688927 10.1152/ajpheart.1998.275.1.H301

[B11] Cressman J. R. Ullah G. Ziburkus J. Schiff S. J. Barreto E. (2009). The influence of sodium and potassium dynamics on excitability, seizures, and the stability of persistent states: I. Single neuron dynamics. J. Comput. Neurosci. 26, 159–170. 10.1007/s10827-008-0132-4 19169801 PMC2704057

[B12] Deb K. (2001). Multi-Objective Optimization Using Evolutionary Algorithms. Available online at: https://www.google.co.il/books?hl=iw&lr=&id=OSTn4GSy2uQC&pgis=1 [Accessed February 5, 2015].

[B13] Eilers J. Augustine G. J. Konnerth A. (1995). Subthreshold synaptic Ca^2+^ signalling in fine dendrites and spines of cerebellar Purkinje neurons. Nature 373, 155–158. 10.1038/373155a0 7816097

[B14] Fierro L. DiPolo R. Llano I. (1998). Intracellular calcium clearance in Purkinje cell somata from rat cerebellar slices. J. Physiol. 510 (Pt. 2), 499–512. 10.1111/j.1469-7793.1998.499bk.x 9705999 PMC2231061

[B15] Fleidervish I. A. Lasser-Ross N. Gutnick M. J. Ross W. N. (2010). Na^+^ imaging reveals little difference in action potential-evoked Na^+^ influx between axon and soma. Nat. Neurosci. 13, 852–860. 10.1038/nn.2574 20543843 PMC3102307

[B16] Forrest M. D. (2014). The sodium-potassium pump is an information processing element in brain computation. Front. Physiol. 5:472. 10.3389/fphys.2014.00472 25566080 PMC4274886

[B17] Forrest M. D. Wall M. J. Press D. A. Feng J. (2012). The sodium-potassium pump controls the intrinsic firing of the cerebellar purkinje neuron. PLoS ONE 7:e51169. 10.1371/journal.pone.0051169 23284664 PMC3527461

[B18] Hay E. Hill S. Schürmann F. Markram H. Segev I. (2011). Models of neocortical layer 5b pyramidal cells capturing a wide range of dendritic and perisomatic active properties. PLoS Comput. Biol. 7:e1002107. 10.1371/journal.pcbi.1002107 21829333 PMC3145650

[B19] Hay E. Segev I. (2015). Dendritic excitability and gain control in recurrent cortical microcircuits. Cereb. Cortex 25, 3561–3571. 10.1093/cercor/bhu200 25205662 PMC4585504

[B20] Hines M. L. Carnevale N. T. (1997). The NEURON simulation environment. Neural Comput. 9, 1179–1209. 10.1162/neco.1997.9.6.1179 9248061

[B21] Hines M. L. Davison A. P. Muller E. (2009). NEURON and python. Front. Neuroinform. 3:1. 10.3389/neuro.11.001.2009 19198661 PMC2636686

[B22] Hines M. L. Morse T. Migliore M. Carnevale N. T. Shepherd G. M. (2004). ModelDB: a database to support computational neuroscience. J. Comput. Neurosci. 17, 7–11. 10.1023/B:JCNS.0000023869.22017.2e 15218350 PMC3732827

[B23] Jeon D. Yang Y.-M. Jeong M.-J. Philipson K. D. Rhim H. Shin H.-S. (2003). Enhanced learning and memory in mice lacking Na^+^/Ca^2+^ Exchanger 2. Neuron 38, 965–976. 10.1016/S0896-6273(03)00334-9 12818181

[B24] Kaczmarek L. K. (2013). Slack, slick and sodium-activated potassium channels. ISRN Neurosci. 2013:354262. 10.1155/2013/354262 24319675 PMC3850776

[B25] Kiedrowski L. Brooker G. Costa E. Wroblewski J. T. Andreeva N. Khodorov B.. (1994). Glutamate impairs neuronal calcium extrusion while reducing sodium gradient. Neuron 12, 295–300. 10.1016/0896-6273(94)90272-0 7906528

[B26] Konnerth A. Takechi H. Eilers J. (1998). A new class of synaptic response involving calcium release in dendritic spines. Nature 396, 757–760. 10.1038/25547 9874373

[B27] Korngreen A. Kaiser K. M. M. Zilberter Y. (2005). Subthreshold inactivation of voltage-gated K^+^ channels modulates action potentials in neocortical bitufted interneurones from rats. J. Physiol. 562, 421–437. 10.1113/jphysiol.2004.077032 15539396 PMC1665511

[B28] Larkum M. E. Zhu J. J. Sakmann B. (1999). A new cellular mechanism for coupling inputs arriving at different cortical layers. Nature 398, 338–341. 10.1038/18686 10192334

[B29] Larkum M. E. Zhu J. J. Sakmann B. (2001). Dendritic mechanisms underlying the coupling of the dendritic with the axonal action potential initiation zone of adult rat layer 5 pyramidal neurons. J. Physiol. 533, 447–466. 10.1111/j.1469-7793.2001.0447a.x 11389204 PMC2278642

[B30] Lazarewicz M. T. Migliore M. Ascoli G. A. (2002). A new bursting model of CA3 pyramidal cell physiology suggests multiple locations for spike initiation. Biosystems 67, 129–137. 10.1016/S0303-2647(02)00071-0 12459292

[B31] Le Bé J.-V. Silberberg G. Wang Y. Markram H. (2007). Morphological, electrophysiological, and synaptic properties of corticocallosal pyramidal cells in the neonatal rat neocortex. Cereb. Cortex 17, 2204–2213. 10.1093/cercor/bhl127 17124287

[B32] Llinás R. Sugimori M. (1980). Electrophysiological properties of *in vitro* Purkinje cell somata in mammalian cerebellar slices. J. Physiol. 305, 171–195. 10.1113/jphysiol.1980.sp013357 7441552 PMC1282966

[B33] Ma J. Lowe G. (2004). Action potential backpropagation and multiglomerular signaling in the rat vomeronasal system. J. Neurosci. 24, 9341–9352. 10.1523/JNEUROSCI.1782-04.2004 15496670 PMC6730108

[B34] Mainen Z. F. Sejnowski T. J. (1996). Influence of dendritic structure on firing pattern in model neocortical neurons. Nature 382, 363–366. 10.1038/382363a0 8684467

[B35] Martin K. C. Kosik K. S. (2002). Synaptic tagging — who's it? Nat. Rev. Neurosci. 3, 813–820. 10.1038/nrn942 12360325

[B36] Masoli S. Solinas S. D'Angelo E. (2015). Action potential processing in a detailed Purkinje cell model reveals a critical role for axonal compartmentalization. Front. Cell. Neurosci. 9:47. 10.3389/fncel.2015.00047 25759640 PMC4338753

[B37] Meurer A. Smith C. P. Paprocki M. Čertík O. Kirpichev S. B. Rocklin M. (2017). SymPy: symbolic computing in Python. PeerJ Comput. Sci. 3:e103 10.7717/peerj-cs.103

[B38] Mondragão M. A. Schmidt H. Kleinhans C. Langer J. Kafitz K. W. Rose C. R. (2016). Extrusion versus diffusion: mechanisms for recovery from sodium loads in mouse CA1 pyramidal neurons. J. Physiol. 594, 5507–5527. 10.1113/JP272431 27080107 PMC5043027

[B39] Moody W. J. Futamachi K. J. Prince D. A. (1974). Extracellular potassium activity during epileptogenesis. Exp. Neurol. 42, 248–263. 10.1016/0014-4886(74)90023-5 4824976

[B40] Myatt D. R. Hadlington T. Ascoli G. A. Nasuto S. J. (2012). Neuromantic - from semi-manual to semi-automatic reconstruction of neuron morphology. Front. Neuroinform. 6:4. 10.3389/fninf.2012.00004 22438842 PMC3305991

[B41] Powell K. Mathy A. Duguid I. Häusser M. Arenz A. Silver R.. (2015). Synaptic representation of locomotion in single cerebellar granule cells. Elife 4, 977–980. 10.7554/eLife.07290 26083712 PMC4499793

[B42] Pulver S. R. Griffith L. C. (2010). Spike integration and cellular memory in a rhythmic network from Na^+^/K^+^ pump current dynamics. Nat. Neurosci. 13, 53–59. 10.1038/nn.2444 19966842 PMC2839136

[B43] Rah J.-C. Bas E. Colonell J. Mishchenko Y. Karsh B. Fetter R. D.. (2013). Thalamocortical input onto layer 5 pyramidal neurons measured using quantitative large-scale array tomography. Front. Neural Circuits 7:177. 10.3389/fncir.2013.00177 24273494 PMC3824245

[B44] Rapp M. Segev I. Yarom Y. (1994). Physiology, morphology and detailed passive models of guinea-pig cerebellar Purkinje cells. J. Physiol. 474, 101–118. 10.1113/jphysiol.1994.sp020006 8014888 PMC1160299

[B45] Rose C. R. Konnerth A. (2001). NMDA receptor-mediated Na^+^ signals in spines and dendrites. J. Neurosci. 21, 4207–4214. 11404406 10.1523/JNEUROSCI.21-12-04207.2001 PMC6762772

[B46] Rose C. R. Kovalchuk Y. Eilers J. Konnerth A. (1999). Two-photon Na^+^ imaging in spines and fine dendrites of central neurons. Pflügers Arch. Eur. J. Physiol. 439, 201–207. 10651018 10.1007/s004249900123

[B47] Santamaria F. Wils S. De Schutter E. Augustine G. J. (2011). The diffusional properties of dendrites depend on the density of dendritic spines. Eur. J. Neurosci. 34, 561–568. 10.1111/j.1460-9568.2011.07785.x 21771115 PMC3156966

[B48] Schäfer C. (2001). Role of the reverse mode of the Na^+^/Ca^2+^ exchanger in reoxygenation-induced cardiomyocyte injury. Cardiovasc. Res. 51, 241–250. 10.1016/S0008-6363(01)00282-6 11470463

[B49] Scheuss V. Yasuda R. Sobczyk A. Svoboda K. (2006). Nonlinear [Ca^2+^] signaling in dendrites and spines caused by activity-dependent depression of Ca^2+^ extrusion. J. Neurosci. 26, 8183–8194. 10.1523/JNEUROSCI.1962-06.2006 16885232 PMC6673787

[B50] Spruston N. Jonas P. Sakmann B. (1995). Dendritic glutamate receptor channels in rat hippocampal CA3 and CA1 pyramidal neurons. J. Physiol. 482, 325–352. 10.1113/jphysiol.1995.sp020521 7536248 PMC1157732

[B51] Swensen A. M. Bean B. P. (2003). Ionic mechanisms of burst firing in dissociated Purkinje neurons. J. Neurosci. 23, 9650–9663. 14573545 10.1523/JNEUROSCI.23-29-09650.2003 PMC6740460

[B52] Swietach P. Spitzer K. W. Vaughan-Jones R. D. (2015). Na^+^ ions as spatial intracellular messengers for co-ordinating Ca^2+^ signals during pH heterogeneity in cardiomyocytes. Cardiovasc. Res. 105, 171–181. 10.1093/cvr/cvu251 25514933 PMC4297422

[B53] Wadiche J. I. Jahr C. E. Regehr W. G. Sakaitani M. Shigeri Y. Yumoto N.. (2001). Multivesicular release at climbing fiber-Purkinje cell synapses. Neuron 32, 301–313. 10.1016/S0896-6273(01)00488-3 11683999

[B54] Wagner S. Castel M. Gainer H. Yarom Y. (1997). GABA in the mammalian suprachiasmatic nucleus and its role in diurnal rhythmicity. Nature 387, 598–603. 10.1038/42468 9177347

[B55] Yarom Y. Spira M. E. (1982). Extracellular potassium ions mediate specific neuronal interaction. Science 216, 80–82. 10.1126/science.6278595 6278595

[B56] Zhang H.-Y. Sillar K. T. (2012). Short-term memory of motor network performance via activity-dependent potentiation of Na^+^/K^+^ pump function. Curr. Biol. 22, 526–531. 10.1016/j.cub.2012.01.058 22405867

[B57] Zylbertal A. Kahan A. Ben-Shaul Y. Yarom Y. Wagner S. (2015). Prolonged intracellular Na^+^ dynamics govern electrical activity in accessory olfactory bulb mitral cells. PLoS Biol. 13:e1002319. 10.1371/journal.pbio.1002319 26674618 PMC4684409

[B58] Zylbertal A. Yarom Y. Wagner S. (2017). Synchronous infra-slow bursting in the mouse accessory olfactory bulb emerge from interplay between intrinsic neuronal dynamics and network connectivity. J. Neurosci. 37, 2656–2672. 10.1523/JNEUROSCI.3107-16.2017 28148726 PMC6596634

