# Supplementary material for: The Slow Dynamics of Intracellular Sodium Concentration Increase the Time Window of Neuronal Integration: A Simulation Study
Source: Front Comput Neurosci. 2017 Sep 20;11:85. doi: 10.3389/fncom.2017.00085 (PMC5609115; doi:10.3389/fncom.2017.00085)
Supplement: Supplementary file 1 [file Image1.PDF]

Supplementary Material

The Slow Dynamics of Intracellular Sodium Concentration Increase the Time Window of Neuronal Integration: A Simulation Study

Asaph Zylbertal*, Yosef Yarom, Shlomo Wagner

*** Correspondence:** Asaph Zylbertal: asaph.zylbertal@mail.huji.ac.il

1.1 Supplementary Figures

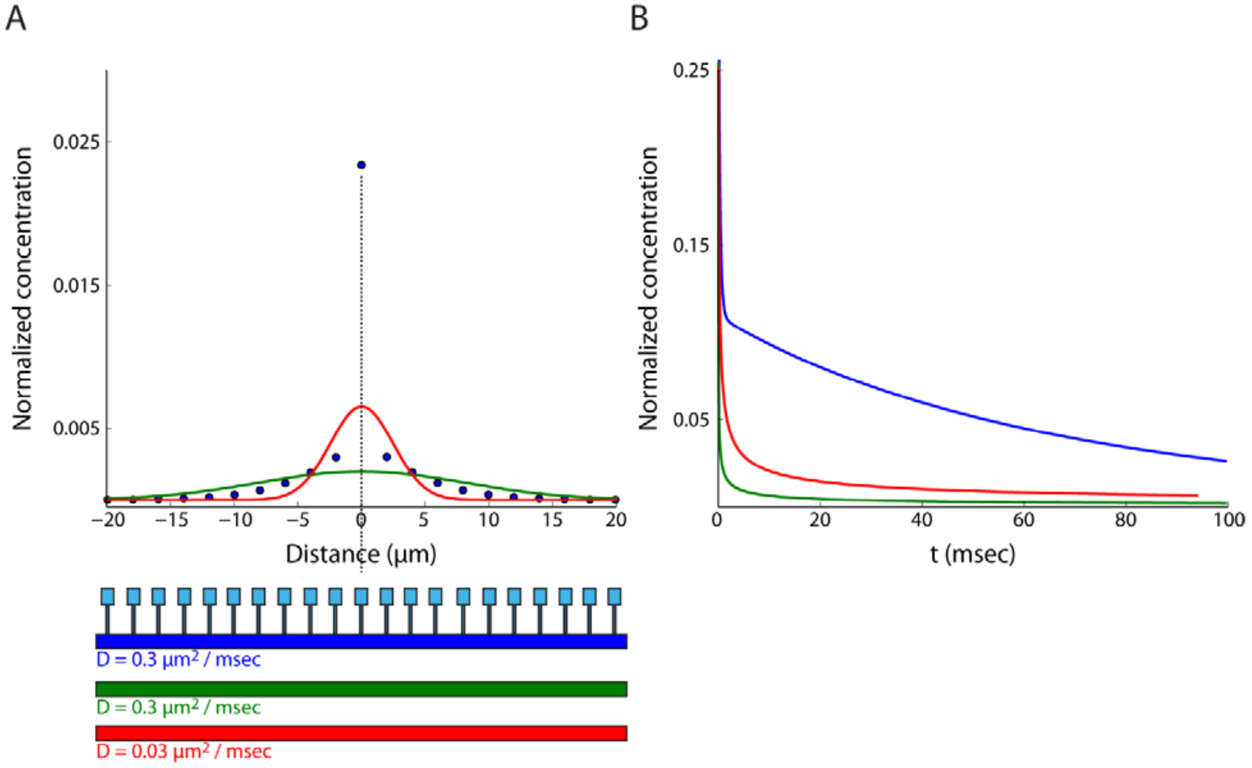

Supplementary Figure. Reducing the diffusion coefficient in a smooth dendrite to 10% of its original value approximates $[Na^+]_i$ dynamics in a spiny dendrite. **A** – Normalized $[Na^+]_i$ as a function of distance from the stimulation site (dotted line), 100 msec following the stimulation, in spine heads (blue dots) or along a smooth dendrite with the original (green) or reduced (red) diffusion coefficient. **B** - The simulated temporal profile of $[Na^+]_i$ (normalized by the initial injected value) in a stimulated dendritic spine embedded within a spiny dendrite (blue), in the point of stimulation in a smooth dendrite (green) and in the point of stimulation in a smooth dendrite where the diffusion coefficient is reduced to 10% of its original value (red). The following morphology was used for this simulation: Dendrite length: 500 µm, dendrite diameter: 1 µm, spine density: 0.5/µm, spine neck length: 1.35 µm, spine neck diameter: 0.25 µm, spine head length and diameter: 0.94 µm.
